# Cell-Type Specific Metabolic Flux Analysis: A Challenge for Metabolic Phenotyping and a Potential Solution in Plants

**DOI:** 10.3390/metabo7040059

**Published:** 2017-11-13

**Authors:** Merja T. Rossi, Monika Kalde, Chaiyakorn Srisakvarakul, Nicholas J. Kruger, R. George Ratcliffe

**Affiliations:** Department of Plant Sciences, University of Oxford, Oxford OX1 3RB, UK; merja.rossi@gmail.com (M.T.R.); moniklde@gmail.com (M.K.); chaiyakorn.s@kbtg.tech (C.S.)

**Keywords:** *Arabidopsis thaliana*, cellular differentiation, green fluorescent protein (GFP), metabolic flux analysis, metabolic phenotype, primary metabolism, reporter protein, systems biology

## Abstract

Stable isotope labelling experiments are used routinely in metabolic flux analysis (MFA) to determine the metabolic phenotype of cells and tissues. A complication arises in multicellular systems because single cell measurements of transcriptomes, proteomes and metabolomes in multicellular organisms suggest that the metabolic phenotype will differ between cell types. In silico analysis of simulated metabolite isotopomer datasets shows that cellular heterogeneity confounds conventional MFA because labelling data averaged over multiple cell types does not necessarily yield averaged flux values. A potential solution to this problem—the use of cell-type specific reporter proteins as a source of cell-type specific labelling data—is proposed and the practicality of implementing this strategy in the roots of *Arabidopsis thaliana* seedlings is explored. A protocol for the immunopurification of ectopically expressed green fluorescent protein (GFP) from *Arabidopsis thaliana* seedlings using a GFP-binding nanobody is developed, and through GC-MS analysis of protein hydrolysates it is established that constitutively expressed GFP reports accurately on the labelling of total protein in root tissues. It is also demonstrated that the constitutive expression of GFP does not perturb metabolism. The principal obstacle to the implementation of the method in tissues with cell-type specific GFP expression is the sensitivity of the GC-MS system.

## 1. Introduction

The metabolic networks that underpin the functions of living cells support an array of intracellular metabolic fluxes that define a metabolic phenotype [1]. The collection of fluxes under a particular set of conditions is often described as a flux map, and the methods available for determining these fluxes include kinetic modelling, constraints-based stoichiometric modelling, and metabolic flux analysis (MFA). All these methods have been used extensively to analyse metabolic networks in plants [2], and MFA is of particular interest because it provides a collection of widely applicable methods for deducing intracellular fluxes that can be assembled into an empirical flux map. Stable isotope labelling experiments, typically using ^13^C-labelled substrates, are central to MFA and the development of the approach in plants [3] followed the establishment of MFA as a routine method for the analysis of micro-organisms [4].

MFA studies of plant and algal cell suspensions are broadly analogous to those of micro-organisms, although with the additional complexity that arises from the extensive subcellular compartmentation of metabolism in such cells [5]. Heterotrophic *Arabidopsis* cell cultures have been used extensively for this purpose [6,7,8,9] as well as algal cells such as *Chlorella protothecoides* [10] and *Chlamydomonas reinhardtii* [11]. The alternative is to conduct the labelling experiments on intact tissues or organs, with numerous studies on cultured oilseeds [12,13,14,15] but also other differentiated systems such as maize root tips [16], hairy root cultures [17,18] and more recently intact *Arabidopsis* rosettes [19,20].

A common feature in all these applications is that the labelling information required for MFA is determined after extraction of the sample, masking potential differences in metabolic phenotype between cell types. While this complication can be ignored in a rapidly dividing and largely de-differentiated cell culture, it is more of a problem in tissues containing multiple cell types. As a specific example, a spatially resolved flux balance analysis of developing *Brassica napus* seeds showed significant differences between three tissue types in the developing embryo [21]. More generally, cell-type specific analysis of transcripts [22], proteins [23] and metabolites [24] in roots highlights the molecular heterogeneity of differentiated cells, increasing the likelihood of differences in metabolic flux phenotype.

Recently, there have been substantial advances in the development of single-cell analytical techniques [25,26], including methods for the single cell detection of metabolites in plants [27,28,29]. However, it is not clear that any of the reported methods are capable of providing accurate and precise cell-type specific measurements of the metabolite labelling patterns required for cell-type specific MFA (csMFA). For example, spatially resolved metabolic information can be obtained from intact systems using MS [30,31] or NMR [32] imaging, but MS techniques have not yet been shown to have the capacity to quantify the mass isotopomer distributions (MIDs) of spatially detected metabolites, and NMR imaging is restricted to the detection of only the most abundant metabolites. Isolating particular cell types by flow cytometry is another promising technique [29] but the length of the procedure is likely to lead to perturbations in the metabolic phenotype of the target cells during cell sorting.

An alternative approach for obtaining cell-type specific labelling data could be to purify proteins whose expression is restricted to a specific cell type from total tissue extracts. The labelling patterns of the amino acids in protein hydrolysates are routinely used in constructing flux maps of primary metabolism, and a reporter protein from a particular cell type might provide sufficient data for csMFA. The reporter protein could be endogenously expressed, or it could be the result of targeting a potentially inducible transgene to the tissue of interest. In support of the former suggestion, subcellular information on heterotrophic metabolism in plastids is routinely obtained from the starch in whole cell extracts [5], and differences in the labelling of the two subunits of rubisco have been interpreted in terms of the cytosolic and plastidic locations for their synthesis [33]. While there may be obvious candidates for cell-specific reporter proteins in particular instances, for example nitrogenase as a reporter for the bacteroids in a root nodule, or photosystem I in co-cultures of heterotrophic and photoautotrophic microorganisms [34], a more flexible approach would be to transform the system with a gene construct encoding a foreign protein under the control of a cell-type specific promoter. Green fluorescent protein (GFP) has already been used as a reporter protein for MFA of bacterial cells [35,36] and *Arabidopsis* lines with cell-type specific expression of GFP in roots are available [37].

Here, the confounding effect of cellular heterogeneity on conventional MFA is demonstrated using computational analysis and the potential application of GFP as a reporter protein for csMFA explored. A protocol for the immunopurification of GFP from the roots of *Arabidopsis thaliana* seedlings is developed. This procedure is used to establish that constitutively expressed GFP reports accurately on the labelling of total protein in root tissues and that constitutive expression of GFP does not perturb metabolism, thereby laying the foundations for the use of this reporter protein for csMFA in plants.

## 2. Results

### 2.1. Confounding Effect of Cellular Heterogeneity on Metabolic Flux Analysis

**Figure 1:**
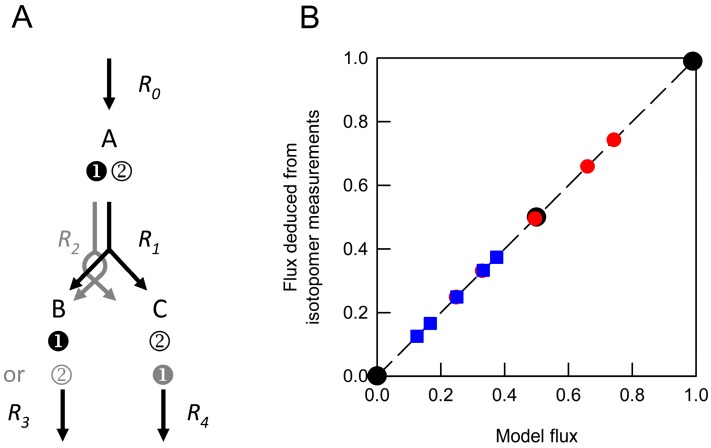
Reliability of flux estimates in a network containing a single variable flux. (**A**) Model network in which carbon atoms in intermediate A are transferred to products B and C in different ways through the activities of alternative irreversible reactions (*R*_1_ and *R*_2_). The isotopomer compositions of the metabolic intermediates B and C were determined when flux through *R_1_* was varied. (**B**) Fluxes were deduced from individual isotopomer datasets (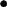) and from weighted average datasets generated by combining values obtained from networks in which *R*_1_*net* was 0 and 1.0 (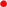) or 0 and 0.5 (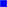). These were compared with the anticipated model flux based on the relative contributions of different networks to the isotopomer dataset. Each deduced flux value is the mean of 100 Monte Carlo simulations for which the SD is smaller than the symbol. The dashed line indicates equivalence between the deduced flux estimate and model flux.

The effect of cellular heterogeneity on the analysis of steady-state labelling patterns can be investigated in silico using simple model networks. Consider a system of two cell types with the same metabolic network supporting different flux distributions. The specific question to be addressed is whether the measured isotopomer abundances from such a system, which would be weighted averages of the unknown values for the two cell types, yield accurate estimates of the averaged fluxes through the network. Figure 1 shows that for a simple network defined by a single flux (Table S1), averaged isotopomer abundances do indeed yield an accurate estimate of the weighted flux.

**Figure 2:**
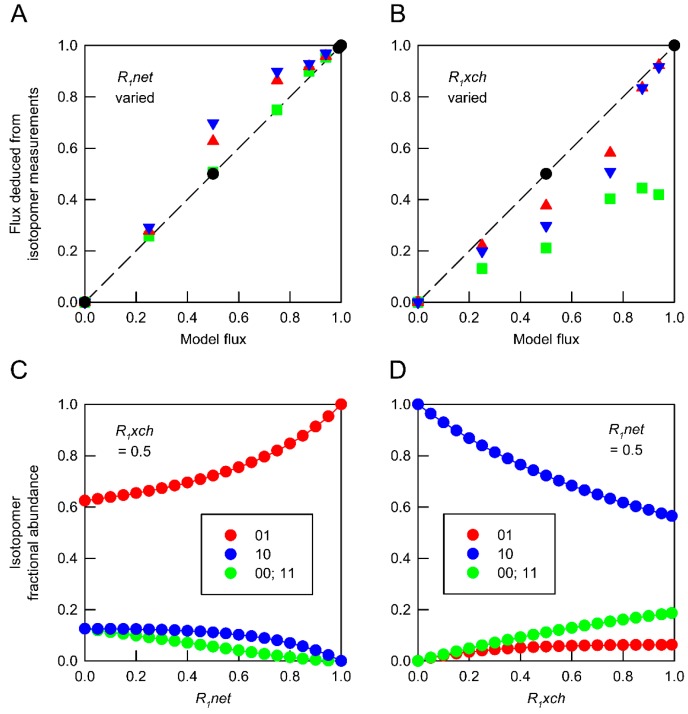
Reliability of flux estimates in a network containing two variable fluxes. The basic model described in Figure 1 was modified by making *R_1_* reversible. Flux through this network is defined by a single net flux (*R_1_net*) and a single exchange flux (*R_1_xch*). Simulated isotopomer measurements for metabolites (**A**–**C**) were obtained for networks in which both of these fluxes were varied independently. Fluxes deduced from individual isotopomer datasets and from weighted average datasets generated by combining values obtained from different networks were compared with the anticipated model flux determined by the relative contributions of the different networks to the isotopomer dataset. (**A**) Deduced values of *R_1_net* from combinations of networks in which *R_1_xch* values were held at 0 (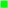), 0.5 (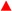) and 0.99 (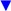). (**B**) Deduced values of *R_1_xch* from combinations of networks in which *R_1_net* values were held at 0 (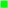), 0.5 (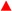) and 1.0 (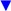). In both panels, isotopomer datasets from individual networks yielded the expected flux values under all conditions (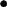). Each deduced flux value is the mean of 100 Monte Carlo simulations for which the SD is smaller than the symbol. The dashed line indicates equivalence between deduced flux estimate and model flux. The influence of flux distribution on the isotopomer composition of metabolic intermediates is exemplified by changes in the composition of metabolite A in response to (**C**) varying *R_1_net* while *R_1_xch* is held at 0.5, and (**D**) varying *R_1_net* while *R_1_xch* is held at 0.5. Isotopomers are identified using 0 to indicate ^12^C and 1 to indicate ^13^C in specified positions. In these simulations, the fractional abundances of 00 and 11 are identical.

However, increasing the complexity of the network by the addition of a second independent flux (Table S1) leads to a situation in which averaged isotopomer values do not provide accurate estimates of the weighted fluxes (Figure 2A,B). The reason for this is the non-linear relationship between the flux values and the fractional abundances of the isotopomers (Figure 2C,D). In the simple model (Figure 1A), the distribution of the label in B and C is directly proportional to the relative flux through the two routes for the metabolism of A leading to accurate estimates of the averaged flux. In contrast, with two independent (non-zero) fluxes, the labelling of a particular isotopomer is not directly proportional to the fluxes (Figure 2C,D) and so averaged isotopomer data cannot be expected to produce the correct averaged flux values.

The effect of cellular heterogeneity in a biologically more realistic system was investigated using a model representing the canonical uncompartmented network of primary carbon metabolism, comprising glycolysis, the oxidative pentose phosphate pathway, the tricarboxylic acid cycle, and the glyoxylate cycle, with relevant outputs for major classes of biomass components including protein, lipid, cell wall carbohydrate and nucleotides (Table S2). The net fluxes within this network are completely defined by the rate of glucose consumption, the rate of biomass production, and the rates of the internal steps catalysed by glucose 6-phosphate dehydrogenase (G6PDH), phospho-enolpyruvate carboxylase (PEPCase) and isocitrate lyase (ICL). Labelling datasets arising from the metabolism of a combination of 80% [1-^13^C]glucose and 20% [^13^C_6_]glucose in networks with different flux distributions were calculated for 27 fragments from 12 protein-derived amino acids (Table S3) representative of those routinely used in experimental flux analysis [38]. In this way, mass isotopomer distributions were generated for a network representative of *Escherichia coli* under aerobic conditions, and for networks in which either internal fluxes were varied but fluxes to biomass outputs were held constant, or internal fluxes and output fluxes were both varied with the biomass composition held constant or varied (Table S4). We confirmed that the predicted mass isotopomer distributions of this selected subset of metabolite fragments were sufficient to accurately and precisely define the net fluxes through each of the simulated networks (Table S5), albeit with the usual lack of precision for the anaplerotic reactions [39].

The extent to which labelling data obtained from mixtures of two cell types with different flux distributions reflected the overall metabolic activity of the system was investigated by estimating the flux distribution needed to account for the mean mass isotopomer distributions. For each comparison, flux combinations were chosen to yield the same averaged flux across the combined network (Table S4). Table 1 compares the fluxes deduced from the averaged labelling patterns with the expected values of the weighted fluxes that define the overall flux distribution through the network. It is clear, as expected from the results for the simple models (Figure 1 and Figure 2), that the use of labelling data averaged over cells with different metabolic phenotypes, does not necessarily lead to reliable estimates of the aggregate metabolic activity, and more extensive analysis of the system confirmed this conclusion (Table S6).

Table 1 shows that although averaged labelling data can produce seemingly well-defined flux solutions, there can be a substantial discrepancy between these solutions and the true values of the averaged fluxes. Moreover, there is no consistent pattern in the direction of the error, with the predicted fluxes being sometimes greater and sometimes smaller than the true flux. For example, in the simulation in which the internal fluxes differed between the two cell types, the averaged labelling data overestimated the flux through G6PDH and underestimated the fluxes through PEPCase and ICL (Table 1). Similarly, even though the fluxes through these steps were held constant in simulations in which the biomass output of the two cell types was varied, the fluxes deduced from the averaged labelling data differed from the actual values (Table 1). The discrepancies caused by cellular heterogeneity led inevitably to errors in commonly derived physiological parameters such as rates of gas exchange, respiratory quotient and carbon conversion efficiency (Table 1). The effect of cellular heterogeneity on all the fluxes in the network is shown in Table S7, and the conclusion is that the use of labelling data averaged over multiple cell types is not necessarily appropriate for metabolic flux analysis. These results corroborate the expectations of theoretical arguments presented by others [40].

### 2.2. Immunopurification of GFP and MS Analysis of GFP Hydrolysates

The proposed solution to the confounding effect of cellular heterogeneity on the analysis of ^13^C-labelling data is to purify cell-type specific reporter proteins from labelled tissues, followed by hydrolysis and derivatization as a precursor to obtaining cell-type specific labelling data by MS. To be successful, it is essential to minimise contamination of the reporter protein, to obtain sufficient amino acids for analysis, and to optimise the method used to quantify the ^13^C-labelling patterns. The practicality of achieving this objective was explored by developing a protocol for the reliable measurement of mass isotopomer distributions for amino acids obtained from GFP that was expressed constitutively in *Arabidopsis* roots.

A His-tagged GFP binding protein derived from a Camelidae single heavy chain antibody (V_H_H) fragment was expressed in *E. coli* and purified by affinity chromatography using Ni-NTA resin. The purified nanobody was coupled to NHS-activated Sepharose to produce an antibody resin which was then used to purify GFP from root extracts from liquid-cultured *Arabidopsis* seedlings. The purity of the GFP was confirmed by SDS-PAGE and the identity of the protein was verified by peptide mass fingerprinting. The typical yield of GFP from the roots of 30–50 seedlings was about 0.7 µg.

**Figure 3:**
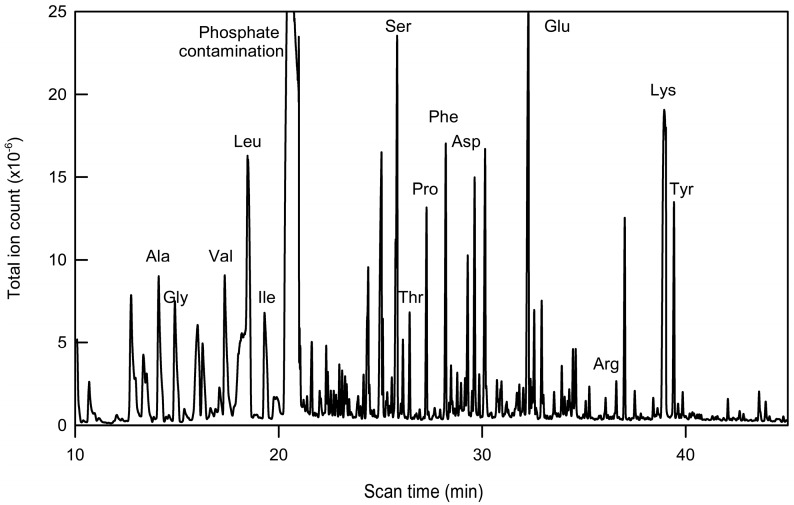
Chromatographic (GC/MS) analysis of amino acids after hydrolysis of GFP isolated from roots of liquid cultured *A. thaliana* seedlings. Immunopurified GFP from three cultures of 30–50 seedlings constitutively expressing the reporter protein was pooled, hydrolysed and derivatised with tBDMS in pyridine. The total ion chromatogram shows peaks from 14 amino acids, as well as many contaminating signals, including phosphate.

Reliable detection and isotopic analysis of the constituent amino acids at these levels of GFP required careful optimisation of the MS analysis. Measurements on amino acid standards of known concentration using an Agilent 7890A GC-MS instrument showed that the majority of the amino acids could be detected at levels above 10^5^ total ion counts in samples containing 20 ng/µL, but that arginine, histidine, cysteine and tryptophan were not detectable at that level even at concentrations of 200 ng/µL. Pooling purified GFP samples and minimising the volume of the derivatization reagent were crucial steps in allowing the majority of the amino acids in a GFP hydrolysate to be detected reliably Figure 3).

The routine interpretation of the spectra from pooled samples was compromised by the presence of non-amino acid contaminants, including phosphate and citrate from the elution buffers used in the purification. This necessitated a further two-step purification process before derivatization: ultrafiltration, which was effective in reducing the citrate level, followed by solid phase extraction filtration to remove the phosphate-derived contaminants. The second step resulted in up to a 30% loss in the total ion counts for the amino acids, but the two purification steps produced samples with negligible levels of contamination.

**Figure 4:**
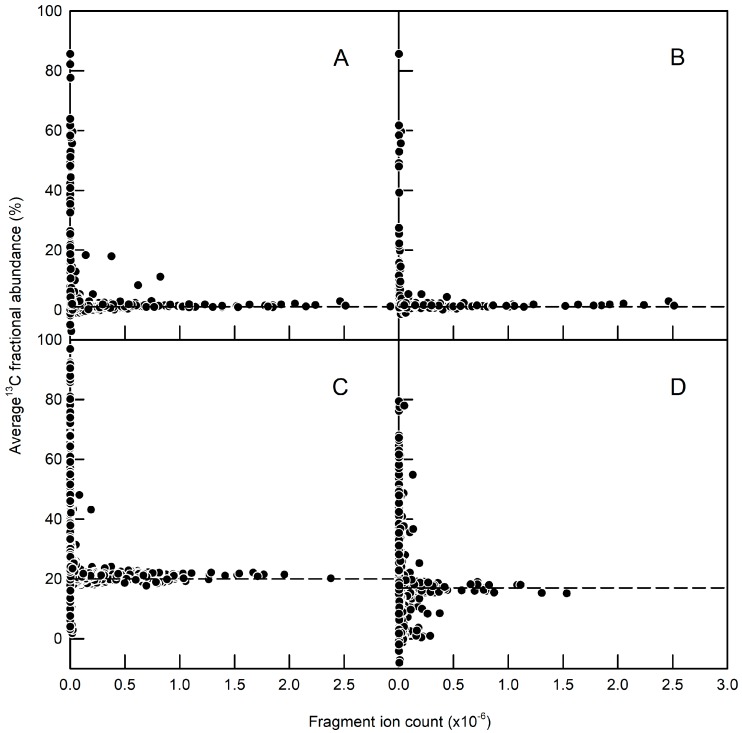
Effect of fragment ion abundance on the measured fractional enrichments of amino acid fragments derived from *A. thaliana* root protein hydrolysates. Seedlings were cultured on either (**A**,**B**) unlabelled glucose, or (**C**,**D**) 20% [^13^C_6_]glucose. (**A**,**C**) Fragments from the total protein fraction obtained from wild-type seedlings and seedlings constitutively expressing GFP. (**B**,**D**) Fragments from immunopurified GFP obtained from seedlings with constitutive GFP expression. Negative values for ^13^C fractional abundances are the result of using a base-line correction algorithm and they highlight the unreliability of signals with low ion counts. In the same way, there are many signals with low fragment ion counts that have infeasibly high ^13^C fractional abundances.

^13^C-MFA depends on the accurate measurement of the MIDs of labelled fragments of amino acids derived from protein hydrolysates. The chromatographic separation and mass spectrometric detection of an amino acid does not in itself guarantee usable MIDs, because of the effect of baseline noise on the quantification of weak signals [41], and this is illustrated in Figure 4, which shows the empirical relationship between calculated fractional abundance and signal intensity for fragments derived from either total protein or GFP isolated from roots of *A. thaliana* seedlings. Plants were cultured on either unlabelled glucose, with ^13^C present at natural abundance (1.1%) or a mixture of unlabelled glucose and 20% [^13^C_6_]glucose. Each dataset shows the same trend with large variations in the measured ^13^C fractional abundance for fragments with low ion counts, and with the expected fractional abundance only being observed reliably for fragment ion counts of at least 10^5^ (Figure 4).

### 2.3. Validation of GFP as a Reporter for the ^13^C Labelling of Total Root Protein in A. thaliana Seedlings with Constitutive GFP Expression

Irrespective of the analytical challenge in quantifying the labelling of amino acid fragments derived from immunopurified GFP, the measured MIDs will only be useful for csMFA if (i) they report accurately on the isotopomer distributions in the amino acid pools that are used for the synthesis of total cellular protein; and (ii) the use of the reporter protein does not perturb primary metabolism in tissues expressing GFP.

**Figure 5:**
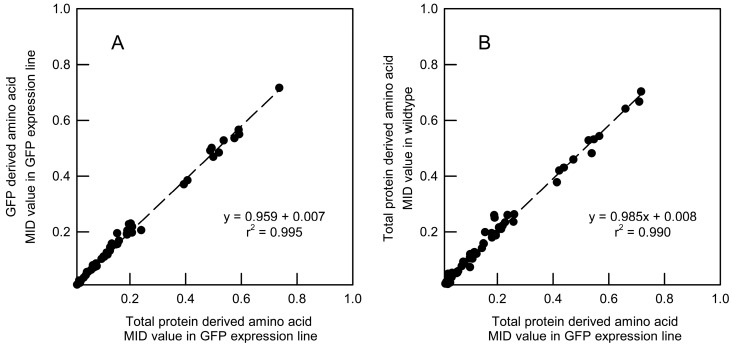
Validation of GFP as a reporter protein for csMFA. (**A**) Comparison of MIDs of amino acids derived from either GFP or total protein obtained from the roots of *A. thaliana* seedlings expressing GFP. The seedlings were grown in 20% [^13^C_6_]glucose and total ion counts measured for amino acid mass peaks were processed into MIDs to obtain average data on each mass peak for each fragment. The plot includes data from 12 amino acids, corresponding to the reliable detection of identical fragments in the two protein fractions. A paired *t*-test established that there was no significant difference between the abundances of individual amino acid isotopomers derived from total protein and GFP (*t*-value = 0.063, d.f. = 68, *p* = 0.950). (**B**) Comparison of total protein MIDs from wild-type and GFP-expressing seedlings. In both plots, the coefficient of linear regression was one with an intercept of zero and the coefficient of determination was close to unity demonstrating the equivalence of the mass isotopomer distributions in the systems under comparison. A paired *t*-test established that there was no significant difference between the abundances of individual amino acid isotopomers derived from total protein in wild-type and the GFP-expressing line (*t*-value = 0.060, d.f. = 59, *p* = 0.953).

To test the first proposition, the MIDs of amino acid fragments derived from GFP purified from *A. thaliana* roots were compared with the MIDs obtained from the total protein fraction from the same tissue (Figure 5A). Not all of the amino acids could be detected reliably in both fractions, but for the 12 amino acids for which reliable data could be obtained from the same fragments it was apparent from the linear regression that the MIDs derived from the reporter protein were indistinguishable from those derived from the total protein fraction (Figure 5A). In agreement with this analysis, all the amino acid fragments were labelled to approximately 20% as expected and there was no significant difference between the labelling of the fragments from the two fractions (Table S8). Thus, the labelling information derived from GFP immunopurified from *Arabidopsis* roots can be used to report on the isotopic labelling in the total protein fraction.

To test whether the reporter protein perturbed primary metabolism, total protein fractions were extracted from the roots of wild-type and GFP expressing seedlings grown in 20% [^13^C_6_]glucose and MIDs were calculated for each detectable amino acid fragment. Comparison between the MIDs for each fragment from the two *Arabidopsis* lines (Figure 5B) provides compelling evidence that flux through primary carbon metabolism in roots is not appreciably affected by expression of GFP at the level achieved in the transgenic plant line.

Overall, the results summarized in Figure 5 demonstrate the suitability of the GFP as a reporter protein for csMFA.

## 3. Discussion

Cellular differentiation arises from changes in gene expression that confer specific functions on particular cell types. In parallel, there are cell-type specific changes in transcriptomes, proteomes and metabolomes that have been well documented, for example, in roots [22,23,24]. It is likely that these changes lead to changes in metabolic phenotype, but the systematic investigation of these changes is hampered by the difficulty of measuring intracellular fluxes at a cellular level. In some instances, changes in metabolic phenotype are likely to be obvious and substantial, for example in C_4_ leaves where the mesophyll and bundle sheath cells make specific contributions to the dominant fluxes associated with photosynthesis [42]. However, more generally, there are many unanswered questions about the extent to which the metabolic network has to adjust to accommodate cell-specific functions and about the metabolic interactions that occur between different cell types. Addressing these questions requires the development of cell-type specific MFA.

The principal conclusion from the computational analysis (Figure 1 and Figure 2; Table 1) is that averaged labelling data from mixtures of different cell types cannot be expected to provide a true representation of the averaged fluxes. This conclusion has its origin in the non-linear relationship between labelling patterns and fluxes, and it implies that flux maps of multicellular tissues derived from averaged labelling data are likely to mask the true contribution of the different cell types. This shortcoming of conventional MFA does not necessarily invalidate comparisons between flux maps for multicellular organisms, but it is a major problem for the interpretation of labelling data at a cell-type specific level in multicellular eukaryotic organisms.

Previous analysis has established that it is possible to resolve metabolic flux distributions within mixed bacterial cultures without prior separation of the bacterial strains (or their components) by fitting labelling data obtained from total biomass to a model in which the metabolic network is duplicated [43], and under favourable circumstances a similar approach could be applied to differentiated tissues. However, in the analysis of bacterial co-cultures, the biomass composition of the component bacterial strains was presumed to be identical, and while this may be a reasonable assumption for two closely related bacterial strains it is unlikely to be true for different cell types in a multicellular tissue. Moreover, the analysis was restricted to a mixture of two strains and it is unclear whether the approach would have sufficient power to resolve fluxes in more complex tissues containing three or more cell types, each with a different flux distribution. It follows that there is a need to develop methods to obtain more informative data that could be used to define metabolic phenotypes of differentiated systems. This is analogous to the resolution of flux through the parallel pathways of carbohydrate oxidation in the cytosol and plastids in plant cells, which is facilitated by analysis of products (in this case sucrose and starch) that report on the labelling patterns of discrete cytosolic and plastidic pools of metabolites [5].

A potential solution to the problem presented by multicellularity is to analyse the labelling patterns of the amino acids derived from a purified, cell-specific reporter protein. While this could be an endogenous protein, a potentially powerful option would be to express an easily purified protein, such as GFP, in a transformed tissue. His-tagged GFP has been used as a reporter for MFA in *E. coli* where it was shown that the labelling patterns for the total protein fraction and purified GFP were equivalent, thus showing that the GFP was synthesized from the same pool of amino acids as the rest of the protein [35]. This was followed by the analysis of co-cultures of wild-type bacteria and GFP-expressing lines and the successful determination of flux ratios from the labelling of GFP [36]. Building on this work, the results reported here show that constitutively expressed GFP can be purified to a high level from the roots of *A. thaliana* seedlings using a procedure based on a GFP-binding nanobody, that the labelling of the GFP matches the labelling of the total protein fraction, and that as expected [35] the expression of GFP at a low level in the transgenic line does not perturb primary metabolism. The lack of any significant metabolic perturbation is also in agreement with the reported absence of any obvious morphological or developmental changes in the GFP-expressing line [44]. The importance of this is that it opens the possibility of exploiting well characterized transformed lines of *A. thaliana* with root cell-type specific expression of GFP [22,37] to determine the metabolic flux phenotypes of specific cell types, such as cortical or endodermal cells.

While these results provide support for the utilization of GFP as a reporter protein for csMFA in roots, the results also highlight a sensitivity issue regarding the amount of GFP required for the accurate and precise determination of the labelling patterns. The GC-MS instrument used for this work was only able to obtain reliable MID measurements from GFP that had been expressed constitutively in roots, and this indicates that the technical challenge of measuring the labelling of GFP expressed in specific cell types will require more sensitive instruments and larger volume injection. A similar sensitivity issue was noted in the GFP analysis of bacterial co-cultures [36], but continuing improvements in MS detector sensitivity suggest that the problem will not be insurmountable. Another option would to use LC-MS/MS to analyse the labelling of peptide fragments [40,45] derived from a tryptic digest of the GFP. This would have the advantage that in principle it would not be necessary to purify the GFP before analysis, but the subsequent extraction of the labelling data for MFA is more complicated than that for the analysis of protein hydrolysates and this method is not yet routine in MFA.

Another issue that will need to be addressed concerns the nature of the data that can be obtained from a reporter expressed in a specific cell type. In conventional MFA, the flux map is usually constrained by information on the identity and labelling of substrates, the labelling of metabolic intermediates and end products—particularly protein and starch in plants—and the allocation of carbon to biosynthetic outputs [6]. In contrast, in csMFA using a reporter protein, it is unlikely that it will be possible to define either the cell inputs or outputs, meaning that these will have to be set as free fluxes in the model. Provided sufficient high quality labelling data are available from the reporter protein, the model will still be over-determined, allowing fluxes to be deduced, but this limitation increases the importance of solving the MS sensitivity issue to ensure that sufficient labelling data is available.

There are two further considerations that will determine the practicality of csMFA using the reporter protein strategy. First, it is not axiomatic that the labelling of the reporter protein will be able to define all the fluxes in the metabolic network. This problem can be explored computationally via the sensitivity matrix that describes the extent to which individual fluxes are constrained by particular measurements [4], and the usual solution is to extend the range of the labelling measurements. For example, in heterotrophic plant cells, knowledge of the labelling of plastidically synthesized starch is required to define the relative contributions of the cytosol and plastid to carbohydrate oxidation [5]. Since this information is unlikely to be available, it seems probable that the definition of flux maps determined by csMFA will be less detailed than those obtained conventionally.

Secondly, intercellular amino acid exchange has the potential to undermine the reporter protein strategy because it is assumed that the reporter protein is synthesized from cell-specific precursors whose labelling reflects the metabolic phenotype of the cell in which it is produced. However, information on the extent of amino acid exchange between cell types is sparse and the extent to which this confounds the method will have to be determined empirically. Interestingly, an analysis in developing *Brassica napus* embryos of the labelling of the large and small subunits of rubisco, which are synthesized in the cytosol and plastid respectively, showed differences in labelling that indicated that some precursor amino acids were not isotopically equilibrated between the two compartments over the timescale of cell growth [33].

Ultimately, the practicality of the reporter protein approach to csMFA will only be established by further experiments, but the confounding effect of cellular heterogeneity on conventional MFA provides sufficient incentive to develop the method further. 

## 4. Materials and Methods

### 4.1. Hydroponic Culture of Arabidopsis thaliana Seedlings

Seeds of *Arabidopsis thaliana* L. (Heynh) ecotype Columbia, and the c-roGFP2 *Arabidopsis* line with constitutive expression of cytosolic GFP [44], were surface sterilised, and then transferred under sterile conditions to conical flasks containing autoclaved growth medium. Half strength Murashige and Skoog medium supplemented with 2.5 mM 2-[N-morpholino]ethanesulfonic acid-KOH (pH 5.7–5.8) and 1% (*w*/*v*) glucose was used as the standard growth medium. Flasks containing 25 mL of medium and 30–50 seeds were placed on an orbital shaker (70–100 rpm) for 2 d in the dark at 4 °C, and then grown with continuous shaking for 14 d at 22 °C in a 16 h/8 h light/dark cycle. For experiments that required isotope labelling, 20% of the glucose in the medium was supplied as [^13^C_6_]glucose. Carbohydrate is the principal respiratory substrate in roots and glucose is therefore commonly used as a source of ^13^C label in steady-state MFA. However, flux maps of plant tissues obtained with this labelling strategy should be interpreted within the context of the increasing evidence for the potential signalling effects of carbohydrates [46].

### 4.2. Extraction of Total Protein

Root systems from 30–50 cultured seedlings were ground into a powder in liquid nitrogen and extracted with 5 mL 100% methanol. The methanol-insoluble material was homogenised in 2 mL of extraction buffer containing 50 mM 4-(2-hydroxyethyl)-1-piperazineethanesulfonic acid (HEPES)-KOH (pH 7.5), 10 mM EDTA, 0.5% (*w*/*v*) PVP, 50 mM NaCl and 1% (*v*/*v*) Triton X-100. The homogenate was centrifuged at 3400× *g* at 4 °C for 10 min in 50 mL tubes to obtain a soluble protein extract.

### 4.3. Expression and Purification of the GFP Binding Protein in Escherichia coli

Competent *E. coli* BL21 cells were transformed with plasmid pHEN6-GBP, a derivative of pHEN6 [47] containing the coding sequence of a high-affinity GFP-binding V_H_H domain isolated from alpaca (*Vicugna pacos*) linked to a periplasm-targetted (pelB) leader sequence and fused to a C-terminal histidine (His_6_) tag [48]. Transformed cells were grown at 37 °C in TB medium supplemented with 2% (*w*/*v*) glucose and 100 µg mL^−1^ ampicillin to an optical density of 0.8–0.9. Expression of GBP was then induced by addition of 1 mM IPTG and the culture incubated overnight at 26 °C. Cells were harvested by centrifugation, subjected to osmotic shock, and GFP binding protein purified from the periplasmic fraction with Ni-NTA resin (Qiagen, Hilden, Germany) and an imidazole elution buffer using the manufacturer’s recommended procedure (The QIAexpressionist: Protocol 11. Preparation of 6xHis-tagged periplasmic proteins from *E. coli*). The protein concentration of purified samples was determined by the Bradford assay using bovine serum albumin as a standard.

### 4.4. Immunopurification of GFP from Plant Protein Extracts

Nanotrap beads were formed by coupling 1.5 mg of GFP binding protein to 1 mL of N-hydroxysuccinimide-Sepharose following the manufacturer’s instructions (GE Life Sciences, Chalfont St Giles, UK).

Root systems from 30–50 cultured seedlings were ground into a powder in liquid nitrogen and the powder was homogenised in approximately two volumes of extraction buffer (Section 4.2) supplemented with 5% (*v*/*v*) glycerol and cOmplete^TM^ Protease Inhibitor Cocktail (Roche, Basel, Switzerland, prepared according to the manufacturer’s instructions). The resulting protein extract was centrifuged at 3400× *g* for 10 min at 4 °C, and the resulting supernatant re-centrifuged at 14,400× *g* for 3 min at 4 °C to provide a clear supernatant.

To purify GFP, 50 μL of the GFP-binding-protein nanotrap resin was added to the clarified plant protein extract in a microfuge tube which was then incubated with shaking for 30 min at 4 °C. Unbound protein remaining in solution was removed by centrifugation (5000× *g* for 3 min at 4 °C), and the resin was washed five times, each time by resuspension in 1 mL of dilution buffer containing 20 mM Tris-Cl (pH 7.5), 300 mM NaCl, 0.5 mM EDTA [49]. The bound GFP was then eluted using 50 μL 100 mM Na-citrate buffer (pH 3.2).

### 4.5. Preparation of GFP Samples for GC-MS

Immunopurified GFP was desalted using an Amicon Ultra 0.5 mL centrifugal filter (Merck Millipore, Burlington, MA, USA) containing an ultrafiltration membrane with a 10 kD exclusion limit. The 50 μL GFP sample was diluted to 0.5 mL with water and then concentrated to its original volume by centrifugation. This dilution and reconcentration of the sample was repeated a further four times. In the final concentration step, the sample was reduced to 20 μL. The desalted protein was then subjected to solid phase extraction using a Ziptip C_18_ (Merck Millipore, Burlington, MA, USA) according to the manufacturer’s instructions. Purified GFP was eluted in acetonitrile, dried and finally redissolved in 50 μL deionised water.

### 4.6. Gas Chromatography—Mass Spectrometry

Total protein or purified GFP was hydrolysed overnight in 0.5 mL of 6 M HCl at 100 °C. The resulting hydrolysate was centrifuged to remove insoluble material, and then neutralized with 1 M KOH. Dried aliquots containing 5–10 µg of total protein hydrolysate were dissolved in 25 µL of pyridine and shaken at 950 rpm for 30 min at 37 °C. Then, 35 µL of MtBSFA (Regis Technologies, Morton Grove, IL, USA) was added and the mixture was incubated with shaking for 30 min at 65 °C. For GFP samples containing 2–3 µg of protein hydrolysate, the volumes of pyridine and MtBSFA were reduced to 8 µL and 12 µL respectively. The derivatized sample was transferred to a 0.2 mL conical crimp neck glass vial (HiChrom, Reading, UK) and subsequently analysed by GC-MS on an Agilent 7890A GC coupled to an Agilent 5975C quadrupole MS using 1 µL sample volumes and procedures described elsewhere [50]. Mass spectra were acquired in scan mode for the range *m/z* 146–600 at a speed of 4.38 scan s^−1^. MetAlign was used for baseline correction [51] and MSCorr was used to correct each detected amino acid fragment for the natural abundance of the carbon atoms derived from the derivatising agent and isotopes of all other elements [52]. Mass isotopomer abundances and fractional enrichment values were determined as before [50].

### 4.7. Metabolic Modelling

Metabolic modelling was conducted using 13C-FLUX (version 20050329) to analyse positional isotopomer data [4] or INCA (version 1.5) to analyse mass isotopomer data [53].

For analysis of the simple theoretical networks, the CumoNet routine of 13C-FLUX was used to establish the steady state isotopomer composition of metabolites following redistribution of label throughout the network defined by the model presented in Figure 1 and its variant described in Figure 2. In these models, which are defined explicitly in the format used by 13C-FLUX in Table S1, flux through the network is defined by the values of *R*_0_*net* (constrained to 1), *R*_1_*net* and *R*_1_*xch* (when active). Positional isotopomer distributions were determined for the three internal metabolites (A–C) at values of *R*_1_*net* over the range 0–1.0 and (where appropriate) *R*_1_*xch* over the range 0–0.99 for networks supplied with substrate labelled exclusively in position 1.

The extent to which labelling information in metabolites defines fluxes throughout the network used to generate the data was examined by obtaining flux solutions using the fractional abundances of all isotopomers of each of the internal metabolites. The free fluxes were fitted to labelling data derived from individual networks or from weighted averages of datasets from different networks using the Donlp2 sequential quadratic programing algorithm. The reliability of flux estimates was determined by Monte Carlo simulations involving 100 replicate determinations for each combination of isotopomer measurements which were all assigned a nominal standard deviation of 1.0% of the measurement value with a minimum absolute value of 0.001.

Analysis of the canonical non-compartmented network of core metabolism was based on mass isotopomer measurements using the model described in Table S2 in the format used by INCA. Steady-state mass isotopomer distributions for defined fragments of amino acids produced by metabolism of a mixture of 80% [1-^13^C]glucose and 20% [^13^C_6_]glucose were determined using the “Tracer simulation” function. Mass isotopomer datasets were obtained for a range of networks in which internal and output fluxes were varied either independently or in combination (Table S4). Flux distributions were fitted to simulated labelling data derived from individual networks or from weighted averages of the mass isotopomer datasets from different networks using the “Parameter optimization” function with all options set at their default values (Tables S5 and S7). The 95% confidence intervals of flux estimates were determined by parameter continuation analysis based on a nominal absolute error of 0.001 for the fractional abundance of all mass isotopomers.

## Figures and Tables

**Table 1:** Accuracy of flux estimates in heterogeneous networks of primary carbon metabolism. Flux maps were determined from mass isotopomer distributions obtained by combining data from metabolic networks yielding identical mean flux distributions. The networks used to generate the mass isotopomer data differed in internal fluxes alone (models 2 + 3; Table S4), or in combination with variation in biomass output (models 4 + 5; Table S4). Values are best-fit flux estimates with the 95% confidence limits shown in parentheses; flux estimates are also expressed relative to the predicted flux obtained from the mean of the networks used to derive the combined mass isotopomer data. Similar analyses of networks in which biomass output varied in both amount and composition (models 6 + 7; Table S4) are presented in Table S6.

Reaction Step	Model Flux	Internal Fluxes Varied		Biomass Output Varied	
		Estimated Flux	%	Estimated Flux	%
[^13^C_6_]glucose input	20	20.53 (20.48–20.57)	102.6	19.98 (19.91–20.02)	99.9
Glucose 6-P dehydrogenase	50	59.26 (59.04–59.47)	118.5	56.48 (52.08–58.09)	113.0
Phosphoenolpyruvate carboxylase	80	61.03 (60.11–62.11)	76.3	136.19 (61.02–137.68)	170.2
Isocitrate lyase	25	13.51 (11.78–15.24)	54.1	25.19 (23.71–26.84)	100.8
Biomass output	7.56	5.89 (5.73–6.06)	78.0	8.79 (8.63–8.94)	116.2
Physiological output	-	-	-	-	-
CO_2_ production	300	366.11 (359.54–372.78)	122.0	251.29 (245.24–257.47)	83.8
O_2_ uptake	283	352.55 (345.59–359.61)	124.8	231.07 (224.67–237.61)	81.8
Respiratory quotient	1.06	1.04 (1.04–1.04)	97.8	1.09 (1.08–1.09)	102.4
Carbon conversion efficiency	0.50	0.39 (0.38–0.42)	78.0	0.58 (0.57–0.63)	116.2

## Supplementary Materials

The following are available online at www.mdpi.com/2218-1989/7/4/59/s1, Table S1: ^13^C-FLUX model used for analysis of simple hypothetical networks (as analysed in Figure 1 and Figure 2), Table S2: Summary of metabolic reaction network used in simulation studies, Table S3: Summary of amino acid fragments used in simulated flux analysis, Table S4: Variations in fluxes defining network models used in simulation studies, Table S5: Summary of deduced fluxes determined from simulated mass isotopomer distributions, Table S6: Accuracy of flux estimates across an extended range of heterogeneous networks of primary carbon metabolism, Table S7: Flux estimates derived by combining isotopomer data from networks supporting variations in specified fluxes, Table S8: Comparison of average ^13^C fractional abundance of amino acid fragments derived from purified GFP and the total protein fraction.
